# Brain networks involved in tactile speed classification of moving dot patterns: the effects of speed and dot periodicity

**DOI:** 10.1038/srep40931

**Published:** 2017-02-01

**Authors:** Jiajia Yang, Ryo Kitada, Takanori Kochiyama, Yinghua Yu, Kai Makita, Yuta Araki, Jinglong Wu, Norihiro Sadato

**Affiliations:** 1 Division of Medical Bioengineering, Graduate School of Natural Science and Technology, Okayama University, Okayama, 700-8530, Japan; 2 Section on Functional Imaging Methods, National Institute of Mental Health, Bethesda, MD 20892, USA; 3 School of Humanities and Social Sciences, Nanyang Technological University, 14 Nanyang Drive, 637332, Singapore; 4 Division of Cerebral Integration, National Institute for Physiological Sciences, Okazaki, 444-8585, Japan; 5 Department of Physiological Sciences, SOKENDAI (The Graduate University for Advanced Studies), Hayama, 240-0193, Japan; 6 ATR Brain Activity Imaging Center, Seika-cho 619-0288, Japan; 7 Institute of Biomedical and Health Sciences, Hiroshima University, Japan; 8 Key Laboratory of Biomimetic Robots and Systems, Ministry of Education, China

## Abstract

Humans are able to judge the speed of an object’s motion by touch. Research has suggested that tactile judgment of speed is influenced by physical properties of the moving object, though the neural mechanisms underlying this process remain poorly understood. In the present study, functional magnetic resonance imaging was used to investigate brain networks that may be involved in tactile speed classification and how such networks may be affected by an object’s texture. Participants were asked to classify the speed of 2-D raised dot patterns passing under their right middle finger. Activity in the parietal operculum, insula, and inferior and superior frontal gyri was positively related to the motion speed of dot patterns. Activity in the postcentral gyrus and superior parietal lobule was sensitive to dot periodicity. Psycho-physiological interaction (PPI) analysis revealed that dot periodicity modulated functional connectivity between the parietal operculum (related to speed) and postcentral gyrus (related to dot periodicity). These results suggest that texture-sensitive activity in the primary somatosensory cortex and superior parietal lobule influences brain networks associated with tactually-extracted motion speed. Such effects may be related to the influence of surface texture on tactile speed judgment.

Humans exhibit a remarkable ability to judge the speed of a moving object through touch alone^1^,^2^, which is critical for creating a mental representation of the object’s velocity and responding to moving objects in the environment. Though this process has been intensively investigated in vision studies^3^,^4^, the neural mechanisms underlying the tactile judgment of speed remain poorly understood^5^.

In order to calculate the speed of an object’s motion, spatial and temporal information must first be acquired, as speed is defined as the distance travelled divided by the duration of the motion. However, the spatial resolution of the skin is not necessarily high: On a two-point discrimination test, the threshold has been reported as approximately 2 mm, while the threshold for a grating orientation discrimination task has been reported as approximately 1.4mm^6^,^7^. Thus, if the point of an object moves across the fingertip (20 mm width on average)^8^, an error of 7–10% should occur in estimating the speed of that point. Moreover, Essick *et al*. have demonstrated that tactile estimation of speed cannot be explained by the ratio of perceived distance travelled (R_TL_) to perceived duration of travel (R_D_) (i.e., R_TL_/R_D_)^1^,^9^,^10^. Rather, they observed that the process is more accurately explained by perceived duration alone, independent of distance travelled (i.e., 1/R_D_), indicating that tactile estimates of speed are not necessarily calculated according to the ratio of spatial and temporal factors. In contrast, the process may rely on tactile cues that co-vary with speed, as long as such cues are sufficient to infer differences in tested speeds (e.g., temporal cues in the study by Essick *et al*.).

A more recent psychophysical study has revealed that tactile estimates of the speed of a moving object are influenced by the physical properties of the object^2^. Researchers examined the impact of surface textures on speed estimates when the surface of raised dot patterns rotated under the participant’s finger, reporting that increases in inter-element spacing influenced the participant’s estimates of speed^2^. The results of this previous study have led us to question the neural mechanisms that allow individuals to detect different textures and how such mechanisms affect the brain network that represents the speed of the same object.

A number of neuroimaging studies have examined the neural substrates involved in tactile motion processing^11^,^12^,^13^,^14^,^15^,^16^,^17^,^18^. These studies have indicated that tactile motion processing involves a distributed brain network including the postcentral gyrus, parietal operculum, posterior parietal lobule, lateral occipito-temporal cortex, and cerebellum. However, as few studies have examined brain activity when participants are required to judge the direction and speed of a moving object^11^,^15^,^16^, the relative contributions of these regions to tactile motion processing remain unclear. Indeed, to the best of our knowledge, only one neuroimaging study has investigated the neural substrates of tactile speed processing^11^. More specifically, Bodegård *et al*. (2000) reported that a distributed set of brain regions including the somatosensory cortices, posterior parietal lobule, and cerebellum are activated during tactile speed discrimination when a brush was rotated across a participant’s palm^11^. However, as activity during the task was compared only with that observed during a rest condition, it remains unclear which region is associated with differences in motion speed.

In addition, a number of previous neuroimaging studies have identified the neural substrates involved in the tactile processing of object texture. In these studies, haptic texture judgments yielded activation of brain regions such as the postcentral gyrus^19^,^20^,^21^,^22^, parietal operculum^19^,^20^,^21^,^22^,^23^,^24^,^25^,^26^,^27^, and posterior parietal lobule^19^,^20^,^24^,^25^. However, to the best of our knowledge, no previous study has examined brain regions that may be sensitive to the periodicity of dot patterns.

In the present study, functional magnetic resonance imaging was used to investigate brain networks that may be involved in tactile speed classification and how such networks may be affected by an object’s texture. We explored two possibilities. First, we investigated whether brain regions previously implicated in speed and texture processing exhibit interaction effects in order to integrate information on speed and texture^28^,^29^. Second, as tactually-perceived object properties may be processed in separate regions of the brain^23^,^26^,^30^, we examined whether brain regions sensitive to dot periodicity affect activity in speed-related brain regions via changes in functional connectivity. Participants engaged in a tactile speed classification task during which an object rotated along their right middle fingers. We manipulated both the speed and periodicity of dot patterns and evaluated functional imaging data obtained during task performance.

## Results

**Figure 1:**
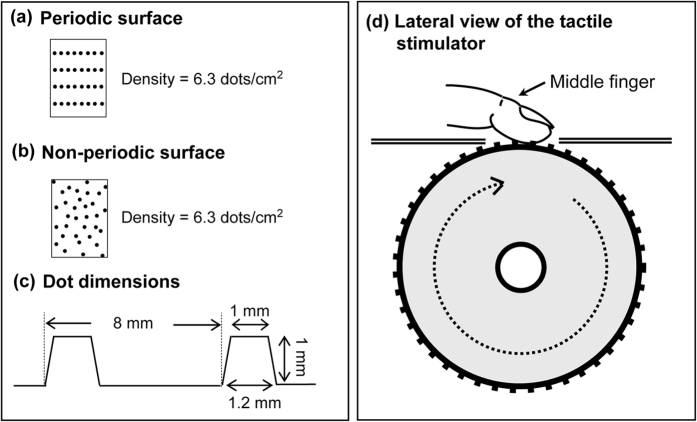
Tactile stimuli. **(a–c)** Configuration of periodic and non-periodic surfaces. Size and number of raised dots was identical between periodic and non-periodic surfaces. The dot dimensions in part **(c)** indicate the inter-element spacing in the direction of the scan for the periodic surface. **(d)** The tactile stimulator. The direction of the scan was proximal to distal, relative to the right middle finger.

We conducted psychophysical (N = 15) and fMRI (N = 20) experiments with healthy participants. In both experiments, the right middle finger of each participant was stimulated using rotating surfaces that contained either periodic or non-periodic dot patterns (Fig. 1). The density of dots and inter-element spacing (in terms of horizontal and vertical axis on the surface) were matched between the two surface types. The speed of moving surfaces ranged between 60 and 140 mm/s.

### Psychophysical Experiment

**Figure 2:**
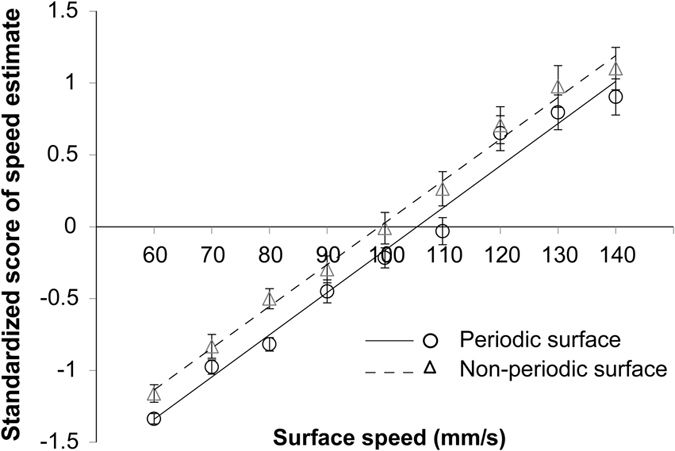
Results of the psychophysical experiment. Standardized subjective estimates of speed are plotted as a function of surface speed. Solid and dashed lines are the regression lines for periodic and non-periodic surfaces, respectively. Data are presented as the mean ± the standard error of the mean (SEM) for 15 participants.

The purpose of the psychophysical experiment was to examine whether estimates of speed differ for periodic and non-periodic surfaces. Participants were required to estimate the speed of moving periodic and non-periodic surfaces by reporting the positive number that best represented their perception of the object’s speed^31^. Mean standardized magnitude estimates are plotted as a function of scanning speed in Fig. 2. Overall, in each surface, mean speed estimates were positively related to the veridical speed. Moreover, the mean speed estimate was greater for periodic than non-periodic surfaces across the range of tested speeds. A two-factor repeated-measures ANOVA (periodicity × speed) revealed significant main effects for speed (F(8, 112) = 196.3, P < 0.001) and dot periodicity (F(1,14) = 6.5, p = 0.02 for dot periodicity) without interaction (P = 0.2).

In order to further validate the main effect of surface type, the linear function was fitted to the data as a function of speed for each surface for each participant. No significant differences were observed between non-periodic and periodic surfaces in the paired *t*-test of the slope of the fitted linear function (P = 0.7), whereas the intercept of the fitted function for the non-periodic surface was greater than the intercept for the function of the periodic surface [t (14) = 2.24, P = 0.04]. Collectively, the motion of non-periodic surfaces was judged to be faster than that of periodic surfaces when surfaces rotated between 60 mm/s and 140 mm/s.

### Functional MRI Experiment

**Figure 3:**
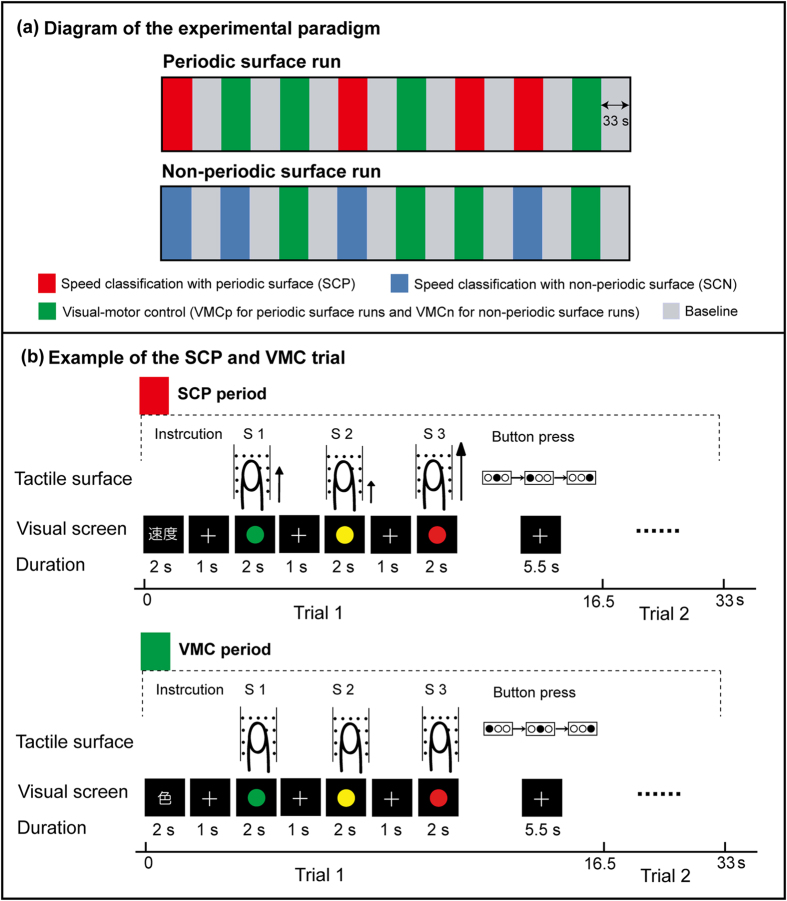
Design of the fMRI experiment. **(a)** Diagram of the experimental paradigm. Each participant engaged in four 540-s runs. Two runs consisted of SCP, VMC, and baseline (rest) conditions (periodic surface runs), while two runs consisted of SCN, VMC, and baseline (rest) conditions (non-periodic surface runs). We inserted a 12-s baseline period prior to the start of the initial period (not shown in the figure). Each period lasted for 33 s. **(b)** Trial. Each period for each task (SCP, SCN, and VMC) consisted of two trials, each of which lasted for 16.5 s. An example of an SCP trial is presented in the upper figure. The participant fixated on the visual cues on the screen. One second after the 2-s visual instruction, a surface rotated across the participant’s right middle finger at three different speeds (S1, S2, and S3). Duration of stimulation at each speed was 2 s, which was alternated with a 1 sec inter-stimulus interval (ISI). Subsequently, the participant classified three speeds using three buttons. The task design of SCN was identical to that of SCP conditions except that a non-periodic surface was used for SCN. Note that colours on the visual screen (green, yellow, and red) were not associated with any speed. The VMC task was different from the other two speed-classification tasks in that the participant classified colours of visual stimuli with their finger contacting static surfaces (lower figure). Thus, VMC was used as a control task for the visual stimuli and response. The BOLD signal during the stimulus presentation at each speed was modelled with each regressor. SCN: speed classification-non-periodic; SCP: speed classification-periodic; VMC: visual-motor control.

During each trial of the fMRI experiment, participants were asked to classify the speed of three consecutively presented periodic surfaces (SCP task) and non-periodic surfaces (SCN task), while ignoring irrelevant visual stimuli (Fig. 3). Participants also engaged in a visual-motor control task designed to control for visual stimuli and response components in the experimental conditions (VMCp for periodic stimuli and VMCn for non-periodic stimuli). In this control task, participants were asked to classify visual stimuli according to colour. The blood-oxygen-level dependent (BOLD) signal during stimulus presentation at each speed was modelled with each regressor (see methods for more details). We applied the same statistical threshold to report brain activation [P < 0.05 at the cluster level, false-discovery rate (FDR) corrected for multiple comparisons over the whole brain, with a height threshold of P < 0.01 uncorrected (two-tailed in t tests)].

#### Behavioural results

##### Performance accuracy

As depicted in Table 1, mean accuracy in all tasks exceeded chance. A one-way repeated measures ANOVA (three tasks) identified a significant main effect of task [F(2, 38) = 23.0; P < 0.001]. Post-hoc pairwise comparisons (using Bonferroni correction) indicated no significant difference between the SCP and SCN tasks; in contrast, the accuracy observed for the VMC task was significantly greater compared with that of the SCP and SCN tasks (P values < 0.001).

##### Response time

Table 1 depicts the mean reaction times for all tasks (relative to the offset of the third stimulus (S3), Fig. 3). A two-way repeated measures ANOVA (three levels of tasks × three response orders) of the mean reaction times revealed significant main effects of task [F(2, 38) = 7.2; P = 0.002] and response order [F (2, 38) = 425.7; P < 0.001], as well as a significant interaction between task and response order [F(4, 76) = 5.9; P < 0.001]. Post-hoc pairwise comparisons (with Bonferroni correction) indicated that response times for the SCP and SCN tasks were significantly longer than those for the VMC task on the first and second stimulus presentations (P < 0.001 for SCP vs. VMC on the first stimulus; P = 0.002 for SCN vs. VMC on the first stimulus; P = 0.01 for SCP vs. VMC on the second stimulus; P = 0.03 for SCN vs. VMC on the second stimulus); however, no significant difference was observed between the SCP and SCN tasks (P values = 1 for all). Collectively, both performance accuracy and response time in the fMRI experiment were comparable between SCN and SCP tasks.

#### Functional MRI Results

##### Speed classification conditions vs. visual-motor control (SCP – VMCp and SCN – VMCn)

Initially, we confirmed that the speed classification tasks (relative to the VMC) activated a widespread set of brain regions including the somatosensory cortices (see Supplementary Figure 1, Supplementary Table 2, and Supplementary Table 3). No significant activation was observed in the lateral occipito-temporal cortex. The opposite contrast [(VMCp – SCP and VMCn – SCN)] revealed significant activation of the bilateral lateral occipito-temporal cortex (Supplementary Table 3).

##### The interaction effect of speed and dot periodicity

The F contrast of the interaction effect between speed and dot periodicity revealed no significant activation.

##### The effect of speed

The F contrast of the main effect of speed showed regions of significant activation in the bilateral superior parietal lobule, precentral gyrus, postcentral gyrus, superior and middle frontal gyrus, angular gyrus, precuneus, supramarginal gyrus, posterior cingulate gyrus, parietal operculum, cuneus, middle and inferior occipital gyrus, superior, temporal and inferior temporal gyrus, putamen, insula, caudate nucleus, hippocampus, parahippocampal gyrus, medial prefrontal cortex, amygdala, orbitofrontal gyrus, lingual gyrus, fusiform gyrus, cerebellum, and brainstem (Supplemental Table 4). In addition, significant activation was observed in the right inferior frontal gyrus and superior occipital gyrus.

**Figure 4:**
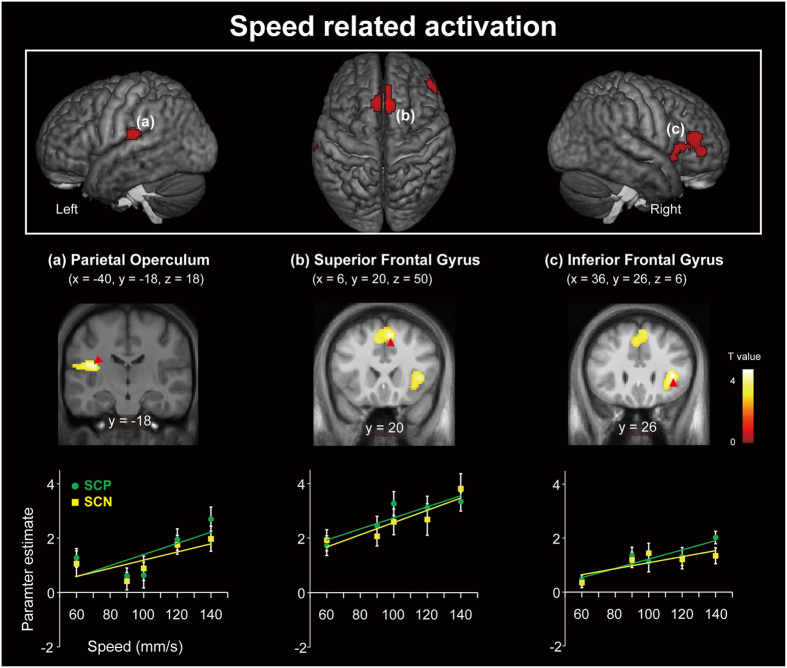
Brain activation positively related to motion speed. Brain regions exhibiting activation positively related to speed in SCP and SCN conditions (Speed+) are presented above. The extent threshold of activation was P < 0.05, FDR corrected for multiple comparisons over the whole brain with height threshold set at P < 0.005 uncorrected (one-tailed). The results were superimposed on a surface-rendered T1-weighted high-resolution MRI averaged across the participants and their coronal sections. Plots indicate activity (parameter estimate) for each surface condition at the peak coordinates in each cluster (Supplementary Table 5). Solid lines represent the regression lines for periodic and non-periodic surfaces, respectively. The error bars indicate the standard error of the mean (SEM). SCP: speed classification-periodic; SCN: speed classification-non-periodic.

We used t-tests to determine which brain regions exhibited graded responses to speed. The t-contrast for activity positively related to speed (Speed+) revealed regions of significant activation in the bilateral superior frontal gyrus, right inferior frontal gyrus, right insula, and left parietal operculum (PO) (Fig. 4 and Supplementary Table 5). A part of the left (contralateral) postcentral gyrus showed a cluster of activation that did not exceed statistical threshold (272 mm^3^). In contrast, the t-contrast for activity negatively related to speed (Speed–) revealed regions of activation in the right (ipsilateral) postcentral gyrus and left cerebellum (Supplementary Table 5).

##### The effect of dot periodicity

**Figure 5:**
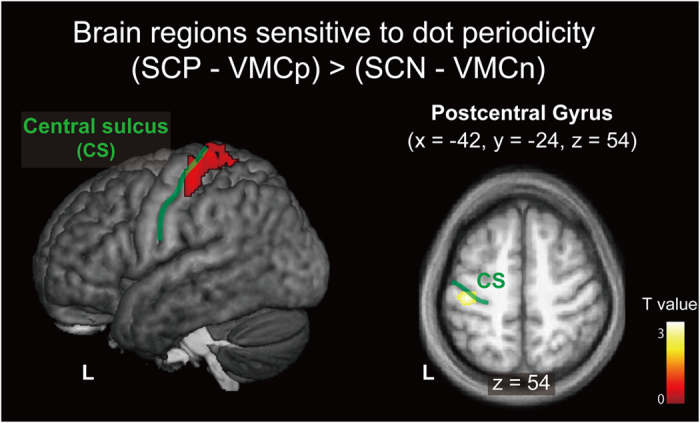
Greater activation produced by the periodic surface than by the non-periodic surface [(SCP–VMCp) – (SCN –VMCn)]. Brain regions exhibiting greater activation for periodic surface tasks than non-periodic surface tasks are depicted above (see also Supplementary Table 6). The extent threshold of activation was P < 0.05, FDR corrected for multiple comparisons over the whole brain with height threshold set at P < 0.005 uncorrected (one-tailed). The results were superimposed on a surface-rendered T1-weighted high-resolution MRI averaged across the participants and their horizontal sections. The solid green line indicates the central sulcus (CS). SCP: speed classification-periodic; SCN: speed classification-non-periodic; VMCp: visual motor control-periodic; VMCn: visual motor control-non-periodic.

We then compared activity in the SCP and SCN in order to determine which brain regions are sensitive to differences between periodic and non-periodic surfaces. As SCP and SCN stimuli were presented in separate runs, we used the VMC conditions as controls. The F test for the effect of dot periodicity revealed significant activation in the left postcentral gyrus and superior parietal lobule (Supplemental Table 4). The subsequent t-contrast of (SCP – VMCp) – (SCN – VMCn) revealed significant activation in the left (contralateral) postcentral gyrus and left superior parietal lobule (Fig. 5 and Supplementary Table 6). The opposite contrast [(SCN – VMCn) – (SCP – VMCp)] revealed no area of significant activation.

##### Overlap between the effects of speed and dot periodicity

We evaluated the overlap of the main effects of speed and dot periodicity determined using F tests. In the left postcentral gyrus and superior parietal lobule, 59% of clusters showing the effect of dot periodicity overlapped with regions showing the effect of speed. Two-way repeated measures ANOVA (5 levels of speed × 2 levels of dot periodicity) of activity in the center of mass of overlap (x = −48, y = −28, z = 60) revealed significant main effects of both dot periodicity (F(1, 19) = 10.2, p = 0.005) and speed (F(4, 76) = 13.1, p < 0.001) without interactions (P = 0.67) (Supplementary Figure 2). One sample t-tests revealed that the mean fitted slopes of linear functions were significantly greater than zero (t(19) = 2.23 p = 0.04 for periodic and t(19) = 2.36 p = 0.03 for non-periodic surfaces).

#### Psycho-physiological Interaction (PPI) analysis

**Figure 6:**
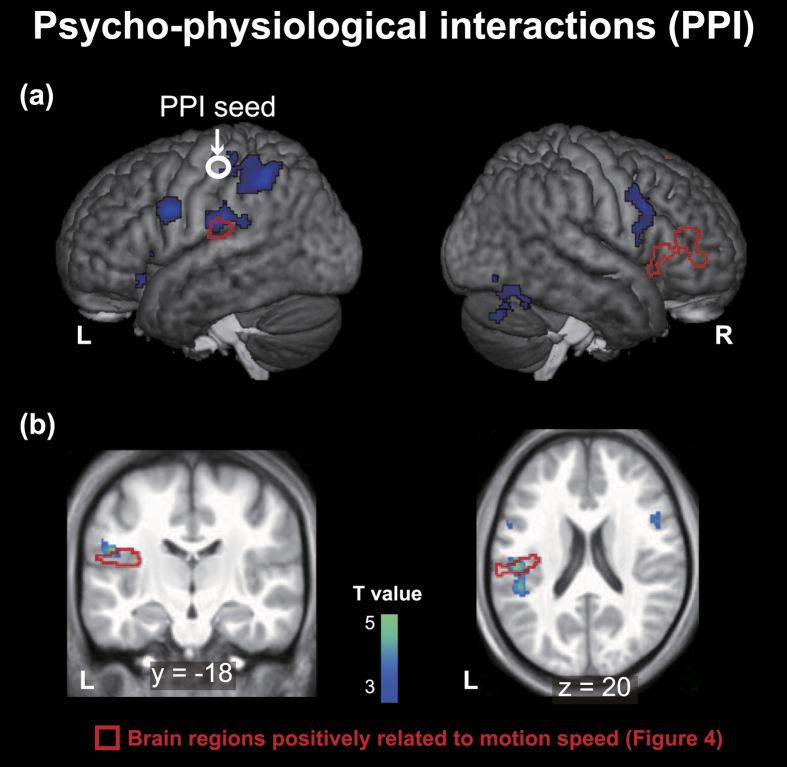
Psycho-physiological interaction (PPI). Context-dependent contributions of the contralateral (left) postcentral gyrus to brain activity in other regions were assessed using psychophysiological interactions (PPI) analysis (see also Supplementary Table 7). The postcentral gyrus, which was activated by dot periodicity (Fig. 5), was defined as a seed region. A part of the PPI effect overlapped with brain regions in which activity was positively associated with motion speed (Fig. 4). The extent threshold of activation was P < 0.05, FDR corrected for multiple comparisons over the whole brain with height threshold set at P < 0.005 uncorrected (one-tailed). Regions surrounded by red lines indicate the speed-related regions depicted in Fig. 4.

We then conducted psycho-physiological interaction (PPI) analysis to further examine whether activity in brain regions sensitive to dot periodicity is related to activity in brain regions associated with speed processing^32^,^33^. In this analysis, the periodicity-sensitive region (i.e., the contralateral postcentral gyrus) was regarded as a seed region, while dot periodicity [(SCP – VMCp) – (SCN – VMCn)] was regarded as a psychological factor^33^. We excluded data of one participant from this analysis because no areas of activation met the criteria of seed selection (see methods for more details). This PPI analysis revealed regions of significant activation in the left superior parietal lobule, right precentral gyrus, left postcentral gyrus, left parietal operculum, left middle frontal gyrus, right lingual gyrus, left lateral orbitofrontal gyrus, left insula, left inferior parietal lobule, right inferior occipital gyrus, bilateral inferior frontal gyrus, right fusiform gyrus, and right cerebellum (Fig. 6 and Supplementary Table 6). These regions of activation overlapped with those whose activity was positively correlated with processing of speed in the contralateral parietal operculum (Fig. 4).

## Discussion

In the psychophysical experiment, participants estimated speed as greater for non-periodic than periodic surfaces when the speed ranged between 60 mm/s and 140 mm/s. A previous study has revealed complex patterns of speed estimates for the two surface types when tested at speeds ranging from 33 to 110 mm/s^2^, though estimated speed tended to be greater for non-periodic than periodic surfaces when speed ranged from 76 to 110 mm/s, consistent with the results of the present study^2^. Therefore, it is possible that the periodicity of dot patterns influences the magnitude of the estimated speed when surfaces move across the finger at relatively high speeds.

Our fMRI results indicate that many brain regions are affected by differences in physical speed, whereas a smaller set of regions such as the parietal operculum, insula, and inferior and superior frontal gyri exhibit responses positively related to physical speed. Activity in the postcentral gyrus and superior parietal lobule was sensitive to dot periodicity. The PPI analysis revealed that dot periodicity modulated functional connectivity between the parietal operculum and postcentral gyrus. These results suggest that dot-periodicity sensitive activity in the parietal cortex influences brain networks associated with tactually-extracted motion speed. To the best of our knowledge, only one previous neuroimaging study demonstrated that tactile speed discrimination (relative to rest) involves a distributed set of brain regions^11^. Here, we extended the findings of this previous study not only by identifying specific brain regions that show graded responses to physical speed, but also by identifying the network implicated in the effect of texture on tactile speed judgment.

One straightforward interpretation of such results is that the regions whose activity was positively related to speed are involved in calculating speed based on its physical definition (distance/time travelled for each dot). However, the calculation is not an easy one to undertake due to the relatively low spatial resolution of the fingertip and the presence of numerous dots on each surface. Essick *et al*. have observed that tactual estimates of speed cannot be fully explained according to this physical definition and are instead more significantly determined by temporal cues^1^,^9^,^10^. Some researchers have suggested that mechanical stimulation itself provides such cues^2^. More specifically, as a surface moves at higher speeds, the fingertip is stimulated by dots with greater frequency. Participants in the present study may have been able to use this temporal frequency cue in order to infer the speed of moving surfaces, a notion consistent with previous findings that activity in the parietal operculum is related to the frequency of vibration^34^,^35^,^36^. In addition to the parietal operculum, we also observed speed-related activation in other brain regions, including the inferior and superior frontal gyrus. Romo *et al*. have demonstrated that a distributed network of brain regions beyond the somatosensory cortices is involved in coding the flutter frequency of vibration provided by tactile stimulation in studies of non-human primates^37^,^38^. Indeed, in order to classify the speed of a moving surface, we must not only encode cues related to motion speed but also memorize and associate such cues with appropriate responses (e.g., button presses). Thus, it is possible that speed-related activation in these regions is further implicated in such cognitive processes of classification.

On the other hand, the contralateral primary somatosensory cortex and superior parietal lobule showed greater activation in periodic surface than non-periodic surfaces. The majority of this region (59%) was modulated by speed and showed a trend of positively graded response. If this region is involved in the process of integrating speed-related cues with periodicity-related cues, we should observe interaction between dot periodicity and speed (e.g., supra-additive effect^29^,^28^). However, no such interaction was observed. In addition, if activity of this region is related to the representation of motion speed, we can expect that this region is sensitive to differences in dot periodicity in the direction of greater estimated speed (Fig. 2). However, activity in these regions was in the opposite direction; non-periodic surfaces exhibited lower activity than periodic surfaces. Therefore, although the primary somatosensory cortex and superior parietal lobule may be associated with dot periodicity and speed, it is unlikely that these regions are involved in the modulation of estimated speed by dot periodicity. Alternatively, as psychophysiological interaction effects were observed between the postcentral gyrus and parietal operculum, our results suggest that the interaction between these somatosensory regions is implicated in the influence of dot periodicity on tactile speed judgment.

Previous studies have indicated that the primary somatosensory cortex and posterior parietal lobule are not only active during tactile texture perception (relative to rest)^19^,^20^,^21^,^22^,^24^,^25^, but that these regions are also sensitive to differences in dot density^27^ and the orientation of linear gratings^25^. Our results are consistent with these findings in that these regions are sensitive to differences in some spatial aspects of textures. The critical difference between periodic and non-periodic surfaces is that dots of periodic surfaces constitute lines orthogonal to the scan direction, and each line of dots stimulates the fingertip simultaneously. Dots that simultaneously stimulate the finger can be perceptually grouped together^39^,^40^. Simultaneous stimulation at separate points on the skin can be perceived as a single, more intense stimulus at the central location (tactile funnelling illusion)^41^. Simultaneous stimulation of two fingers, which can induce the funnelling illusion in humans, produces a single focal activation between the regions that were activated by each finger in area 3b of non-human primates^42^. In humans, patterned tactile stimulation (relative to non-patterned stimulation) produces greater activation in the parietal lobe. For instance, Wacker *et al*. (2011) observed that patterned stimulation produced by dot arrays on the finger activates the posterior parietal lobule and the postcentral gyrus more than randomized stimulation using the same dot arrays^17^. In-phase object motion across two adjacent fingers activates the posterior parietal lobule to a greater extent than out-of-phase motion across the same finger^39^,^40^. Accordingly, it is possible that the sensory inputs of simultaneous dot stimulation are grouped together in the posterior parietal lobule as well as in the somatosensory cortices. Such effects might influence the brain network involved in the processing of speed-related information for dot patterns, resulting in lower estimated speeds for periodic than non-periodic surfaces. It is necessary to validate this speculation in future studies.

In the present study, the lateral occipito-temporal cortex was deactivated during tactile speed tasks (relative to the visual-motor control condition). This result is apparently contradictory to the previous finding that the lateral occipito-temporal cortex is activated by tactile motion stimulation^12^,^13^,^14^,^17^,^18^. However, the present study differs from these studies in that participants of the present study judged the properties pertaining to velocity, while participants in previous studies merely attended to stimuli or detected rare events of tactile stimuli (catch trials). The findings of the present study are consistent with those of a few neuroimaging studies in which participants were required to make judgments regarding the velocity of motion. In these studies, the lateral occipito-temporal cortex exhibited decreased or absent activation in sighted individuals^11^,^15^,^16^. However, it is somewhat puzzling that repetitive transcranial magnetic stimulation (rTMS) of the lateral occipito-temporal cortex was observed to interfere with tactual detection of changes in speed – a fact that remains inconsistent with neuroimaging findings^43^. Thus, further studies are required in order to determine the role of the lateral occipito-temporal cortex in tactile speed processing.

There are three limitations of note with regard to the interpretation of the results of the present study. First, unlike in our previous study^25^, we did not measure finger force. Though finger force should be measured, previous studies have reported that activity in the primary motor cortex (M1) is associated with increased force exerted by the index finger^44^. As we found no activation in the precentral gyrus, we assumed that activation in the present study (motion speed and dot periodicity) would not be influenced by differences in finger force. Second, we used the same surface (either periodic or non-periodic surface) for individual runs. In order to account for the observed differences between periodic and non-periodic runs, we compared SCP and SCN conditions after evaluating each condition alongside a VMC condition in the same run (i.e., [SCP – VMCp] – [SCN – VMCn]). Thus, it is unlikely that the differential changes in BOLD signal observed between periodic and non-periodic surfaces were obtained solely due to the fact that different runs were used for periodic and non-periodic surfaces. Future studies should confirm that the presentation of different surface types within the same run yields the same result. Finally, motion direction and motion speed are related to one another^45^, even though they have been investigated separately^15^,^16^,^46^. In the future, research should focus on how neural substrates underlying speed-related processing are associated with those underlying direction-related processing in the tactual perception of an object’s motion.

In conclusion, the results of the present study indicate that, while many brain regions are affected by differences in the motion speed of dot patterns, the parietal operculum, insula, and superior and inferior frontal gyrus showed positively graded response. Activity in the postcentral gyrus and superior parietal lobule was sensitive to dot periodicity. Functional connectivity analysis also revealed that dot periodicity modulated functional connectivity between the parietal operculum (related to speed) and postcentral gyrus (related to dot periodicity). These results suggest that texture-sensitive activity in the primary somatosensory cortex and superior parietal lobule influence the brain network that are associated with tactually-extracted motion speed. This influence may be related to the effect of physical properties on tactile speed judgment. Such findings constitute an important step toward further understanding of the neural mechanisms underlying tactile speed judgment.

## Methods

### Participants

Thirty-five healthy volunteers consented to participate in the present study. Fifteen healthy male participants (22.7 ± 1.5 years, mean ± SEM) participated in the psychophysical experiment, whereas the remaining participants (13 males and 7 females, 21.9 ± 2.6 years) completed the subsequent fMRI experiment. None of the participants participated in both experiments. All participants were right-handed^47^. No participants had reported loss of tactile sensation, history of major medical or neurological illness such as epilepsy, significant head trauma, or a lifetime history of alcohol dependence. All participants provided written informed consent prior to their participation in the study. The study protocol was approved by the local medical ethics committee at the Okayama University Hospital and National Institute for Physiological Sciences. All methods were carried out in accordance with the approved guidelines.

### Stimuli

We used two types of tactile surfaces, each of which consisted of cylindrical raised dots. The number of dots and the inter-element spacing were matched between the two surface types (6.3 dots/cm^2^). One textured surface (periodic surface) consisted of rectangular arrays of dots with an identical longitudinal transverse spatial period (8 mm), which corresponded to the direction of the scan (Fig. 1a). In contrast, raised dots on the other surface (non-periodic surface) were pseudo-randomly distributed (Fig. 1b). In both surfaces, the dots were raised by 1.0 mm from a 20.0 mm × 376.0 mm square base and by 1.0 mm diameter on top (Fig. 1c). The surfaces were affixed to an MRI-compatible device programmed to automatically stimulate the participant’s finger at a predetermined speed^48^. The device consisted of two cylindrical drums (376.0 mm circumference) mounted on a drive shaft that was rotated by an ultrasonic motor (Fig. 1d). The surfaces were accessible through two rectangular apertures. The direction of the scan was proximal to distal, relative to the right middle finger.

### Psychophysical Experiment

We examined whether speed estimates differed for periodic and non-periodic surfaces. Surfaces were presented at nine different speeds: 60, 70, 80, 90, 100, 110, 120, 130, and 140 mm/s. Each surface-speed combination was repeated ten times for a total of 180 trials. Periodic and non-periodic surfaces were presented in different task blocks. Seven participants engaged in the non-periodic trials prior to the periodic trials, whereas eight participants engaged in the trials in the reverse order. The order of speeds for each surface type was pseudo-randomized. Blindfolded participants placed their right middle fingertip on the surface through an aperture of the device. When each surface was presented at a specified speed on the participants’ middle fingers, participants were asked to estimate and orally report a positive whole number that best matched the speed of motion. They were not informed regarding the number of speeds or surfaces used. Each participant’s response was standardized.

### Functional MRI Experiment

#### Data acquisition

Functional MRI scans were performed using a 3T Siemens Allegra whole-head system (Siemens, Erlangen, Germany). Standard sequence parameters were used to obtain the functional images as follows: T2*-weighted echo planar imaging; repetition time = 3000 ms; echo time = 30 ms; flip angle = 80°; matrix = 64 × 64; 42 axial slices; field of view: 192 × 192 mm; in-plane resolution: 3.0 × 3.0 mm; and 3.0 mm in thickness with a 0.51 mm interslice gap that covered the whole brain. After the acquisition of functional images, T1-weighted high-resolution anatomical images were obtained (voxel size, 0.9 × 0.9 × 1.0 mm^3^).

We presented the periodic and non-periodic surfaces in separate runs (Fig. 3a). Participants completed four 543-s runs, including two runs for the periodic surfaces and two runs for the non-periodic surfaces. Each run consisted of eight 33-s ON periods that alternated with eight 33-s rest (baseline) periods. We inserted a 12-s baseline period prior to initiation of the initial ON period as well as 3-s baseline period following the end of the final rest period (15-s baseline + eight 33-s ON periods + eight 33-s rest periods = 543 s). The eight periods included four periods of speed classification and four visual-motor control (VMC) periods. The order of the ON periods between the speed classification and visual-motor control periods was pseudo-randomized.

Participants were positioned supine in the MRI tunnel with earplugs and instructed to remain relaxed while fixating on a white cross (viewing angle, 1.4 × 1.4 degrees), which was projected through a mirror onto a screen. Participants were further instructed to extend their right arm (comfortably supported by a cushion) to the device. Participants lightly placed their right middle fingertips on the surface, while the other fingers rested on a plastic frame. The left index, middle, and ring fingers were placed on each of the three buttons of the response box.

#### Tactile Speed Classification Tasks

The present study utilized a revised version of a previously reported grating classification task^25^. Each participant was asked to estimate three levels of speeds after the surfaces moved across their right middle fingers at these speeds. The tasks for the periodic (SCP task) and non-periodic (SCN task) surfaces were identical except for the periodicity of dot patterns. An example of an SCP trial is presented in Fig. 3b. The trial onset was cued by an instruction in Chinese characters for the first 2 s. After a 1-s fixation period (white cross), the participant’s finger was stimulated by the surface moving at three speeds with three visual cues in different colours (green, yellow, and red for each stimulus; viewing angle, 3.0 × 3.0 degrees) for 2 s. The presented speeds were pseudo-randomly chosen from 60, 90, 100, 120, and 140 mm/s (e.g., 140, 60, 100 mm/s) for each trial such that mean speeds were matched between periodic and non-periodic surfaces. The order of the colours was also pseudorandomized to avoid any association between a given colour and a specific tactile speed. The tactile stimulation at each speed was alternated with a 1 sec inter-stimulus interval (ISI). After the presentation of the third speed, participants were asked to press three buttons in succession, which were assigned to three different speeds. Participants pushed the left button for the low speed, the middle button for the middle speed, and the right button for the high speed. Participants were asked to press all buttons accurately within 5.5 s. If the participant pressed all three buttons correctly in a trial, the trial was counted as correct. The total duration of the trial was 16.5 s, and there were two trials in each SCP period.

#### Visual-Motor Control (VMC) Task

The Visual-Motor Control (VMC) task was designed to control for visual input and motor responses during the tactile speed classification tasks. The experimental design was the same as that utilized in the SCP and SCN tasks, except for the instructions given to participants and the absence of tactile stimulation. More specifically, visual stimuli of three colours were presented, whereas the right middle finger contacted static surfaces. Participants were instructed to press three buttons in succession, which were assigned to three different colours (left button for green, middle button for yellow, and right button for red). The total duration of one VMC trial was 16.5 s, and there were two trials in each VMC period.

One 33-s baseline period, in which no stimulus was presented, followed each ON period (Fig. 3a). Participants were instructed to fixate on the white cross and to keep their heads and hands as still as possible.

### Data Processing and Analyses

We used Statistical Parametric Mapping (SPM12)^49^ implemented in MATLAB 7.5 (MathWorks, Natick, MA, US) to process and analyse fMRI data. The first four volumes of each fMRI run were discarded due to unsteady magnetization. Functional images from each run were realigned to the first image and then realigned to the mean image following the initial realignment. Slice-timing correction was then performed to adjust for differences in slice-acquisition times. All realigned images were coregistered to the T1-weighted anatomical image. The T1-weighted anatomical image was normalized to Montreal Neurological Institute (MNI) space using the DARTEL procedure^50^. The parameters from the DARTEL procedure were then applied to each functional image as well as to the T1-weighted anatomical image. The normalized functional images were filtered using a Gaussian kernel of 8 mm full width at half-maximum (FWHM) in the x, y, and z axes.

#### Initial individual analysis

A general linear model (GLM) was fit to the fMRI data for each participant. The blood-oxygen-level dependent (BOLD) signal for all tasks was modelled using box-car functions convolved with the canonical hemodynamic response function. The design matrix of each participant included four runs. Each run included 13 task-related regressors. More specifically, each run included five regressors that modelled the 2-s motion stimulation for each speed (60, 90, 100, 120 and 140 mm/s) in the speed classification task. For the visuo-motor control, we included five regressors, each of which emulated the regressor of each motion speed in the tactile classification task (stimulus onset and duration). Finally, we included one regressor for the 2-s instruction period and two regressors modelling the 5.5-s button-press for each task and control. The time series for each voxel was high-pass filtered at 1/128 Hz. Assuming a first-order autoregressive model, the serial autocorrelation was estimated from the pooled active voxels using a restricted maximum likelihood procedure and was used to whiten the data. Motion-related artefacts were minimized via the incorporation of six parameters (three displacements and three rotations) from the rigid-body realignment stage into each model. For each participant, we evaluated the linear contrasts of each task condition relative to baseline. We then obtained the contrast images that were used for the random-effects group analysis.

#### Random-effects group analysis

We employed a full factorial design to construct a single design matrix involving the SCP, SCN, and two VMC conditions (VMCp for each of the periodic and VMCn for each of the non-periodic runs). All conditions were modelled according to within-subject (dependent) levels. We evaluated the linear contrasts of these conditions. The height threshold (SPM{t} and SPM{F}) were set at P < 0.01 uncorrected. The statistical threshold for the spatial extent test on the clusters was set at P < 0.05 and false discovery rate (FDR) corrected for multiple comparisons over the whole brain^51^. Coordinates in MNI space were labelled according to a probabilistic map^52^ in MNI space. We evaluated the following contrasts, which are further detailed in Supplementary Table 1.

##### Comparison with VMC conditions

In order to confirm activation of the somatosensory cortices during surface stimulation, we evaluated the contrast of mean of the SCP conditions with the mean of the VMCp conditions (SCP – VMCp) and the contrast of mean of the SCP conditions with the mean of the VMCn conditions (SCN – VMCn).

##### Interaction effects between motion speed and dot periodicity

We performed an F test to evaluate interaction effects between motion speed and dot periodicity.

##### Motion speed

We evaluated F contrasts for the main effect of speed. We then performed T-tests to identify areas exhibiting activation that was positively or negatively associated with motion speed (Speed + and Speed–). We used standardized scores of motion speeds as contrast weights, as they were strongly correlated with speed estimates in the psychophysical experiments (r values > 0.98).

##### Dot periodicity

We evaluated an F contrast for the main effect of dot periodicity. We then evaluated the T contrasts of (SCP – VMCp) – (SCN – VMCn) and (SCN – VMCn) – (SCP – VMCp) in order to identify brain regions affected by the periodicity of the presented dot patterns.

#### Psycho-Physiological Interactions (PPI) analysis

We performed a PPI analysis to assess task-dependent contributions of a periodicity-sensitive region (i.e., the contralateral primary somatosensory cortex) to activity in other brain regions^32^,^33^. As SCP and SCN conditions were conducted in separate runs, it was necessary to concatenate the time-series of data across all runs in the individual analysis for comparing time-series data between the two conditions. Accordingly, we constructed a design matrix for each participant in which preprocessed data of all four runs were modelled with the same regressors: twenty-six task-related regressors in total (13 regressors each for periodic and non-periodic runs) and 12 realignment parameters (6 for each run). We conducted the following PPI analysis using this design matrix.

##### Definition of seed regions

We evaluated the contrast of [(SCP – VMCp) – (SCN – VMCn)] and extracted time series data for the contralateral postcentral gyrus that met both anatomical and functional criteria. We first determined the coordinates of the postcentral gyrus in the group analysis by evaluating [(SCP – VMCp) – (SCN – VMCn)] (group maximum). We then searched for the participant-specific maxima that were located within the same anatomical gyrus (i.e., postcentral gyrus) and within 8 mm from the local group maximum (individual maximum). All voxels depicted by the same contrast (at the threshold of p < 0.5, uncorrected) within 8-mm diameter around the individual maximum served as the seed region. Time-series data were then extracted.

##### PPI

We then calculated the PPI terms between the seed region and psychological factors in the following three steps. First, the extracted MR signal from each seed region was deconvolved with the canonical hemodynamic response function (HRF). The resulting time series represented an approximation of neural activity^53^. Second, the neural time series data were centred and multiplied (dot product) by a psychological factor of dot periodicity, a vector coding the contrast of [(SCP – VMCp) – (SCN – VMCn)]. Finally, the interaction time series was convolved with the HRF, representing an interaction variable at the hemodynamic level (PPI term).

##### Individual and group analysis

The design matrix at the individual level included not only the PPI regressor, but also the time series of the seed region, the periodicity effect, and regressors of no interest. We evaluated the linear contrast of the PPI regressor for each participant, and the obtained contrast image was used for subsequent group analysis (one-sample *t*-test). We used the same statistical threshold as the other analyses.

## Additional Information

**How to cite this article**: Yang, J. *et al*. Brain networks involved in tactile speed classification of moving dot patterns: the effects of speed and dot periodicity. *Sci. Rep.* **7**, 40931; doi: 10.1038/srep40931 (2017).

**Publisher's note:** Springer Nature remains neutral with regard to jurisdictional claims in published maps and institutional affiliations.

## Supplementary Material

Supplementary Figures and Tables

## Figures and Tables

**Table 1:** Behavioural performance in the fMRI experiment.

Task	Accuracy (%)	Reaction Time (s)		
		Button 1	Button 2	Button 3
SCP	77.4 ± 3.4	1.094 ± 0.055	1.679 ± 0.061	2.218 ± 0.077
SCN	79.8 ± 4.2	1.107 ± 0.067	1.690 ± 0.072	2.223 ± 0.082
VMC	98.8 ± 0.7	0.904 ± 0.047	1.530 ± 0.058	2.128 ± 0.078

Performance accuracy was calculated based on the number of correct trials. Note that reaction time for each button response was measured relative to the end of the third stimulation (S3 in Fig. 3b). All values are presented as mean ± standard error of the mean (SEM). SCN: speed classification-non-periodic; SCP: speed classification-periodic; VMC: visual-motor control.
