# Supplementary material for: Brain networks involved in tactile speed classification of moving dot patterns: the effects of speed and dot periodicity
Source: Sci Rep. 2017 Feb 1;7:40931. doi: 10.1038/srep40931 (PMC5286508; doi:10.1038/srep40931)
Supplement: Supplementary Figures and Tables [file srep40931-s1.pdf]

Supplementary Information

Brain networks involved in tactile speed classification of moving dot patterns: the effects of speed and dot periodicity

Jiajia Yang, Ryo Kitada*, Takanori Kochiyama, Yinghua Yu, Kai Makita, Yuta Araki, Jinglong Wu, Norihiro Sadato

*To whom correspondence should be addressed. E-mail: kitada@nips.ac.jp

This information includes:

7 Supplemental Tables

2 Supplemental Figures

Supplementary Table 1 Predefined contrasts

SCP					VMCp					SCN					VMCn				
60	90	100	120	140	60	90	100	120	140	60	90	100	120	140	60	90	100	120	140
**T contrasts**																			
c01. SCP - VMCp																			
1	1	1	1	1	-1	-1	-1	-1	-1	0	0	0	0	0	0	0	0	0	0
c02. SCN - VMCn																			
0	0	0	0	0	0	0	0	0	0	1	1	1	1	1	-1	-1	-1	-1	-1
c03. Speed+																			
-1.4	-0.4	-0.1	0.6	1.3	0	0	0	0	0	-1.4	-0.4	-0.1	0.6	1.3	0	0	0	0	0
c04. Speed–																			
1.4	0.4	0.1	-0.6	-1.3	0	0	0	0	0	1.4	0.4	0.1	-0.6	-1.3	0	0	0	0	0
c05. (SCP – VMCp) – (SCN – VMCn)																			
1	1	1	1	1	-1	-1	-1	-1	-1	-1	-1	-1	-1	-1	1	1	1	1	1
c06. (SCN – VMCn) – (SCP – VMCp)																			
-1	-1	-1	-1	-1	1	1	1	1	1	1	1	1	1	1	-1	-1	-1	-1	-1
**F contrasts**																			
c07. Interaction effect																			
1	-1	0	0	0	-1	1	0	0	0	-1	1	0	0	0	1	-1	0	0	0
0	1	-1	0	0	0	-1	1	0	0	0	-1	1	0	0	0	1	-1	0	0
0	0	1	-1	0	0	0	-1	1	0	0	0	-1	1	0	0	0	1	-1	0
0	0	0	1	-1	0	0	0	-1	1	0	0	0	-1	1	0	0	0	1	-1
c08. The main effect of speed																			
1	-1	0	0	0	0	0	0	0	0	1	-1	0	0	0	0	0	0	0	0
0	1	-1	0	0	0	0	0	0	0	0	1	-1	0	0	0	0	0	0	0
0	0	1	-1	0	0	0	0	0	0	0	0	1	-1	0	0	0	0	0	0
0	0	0	1	-1	0	0	0	0	0	0	0	0	1	-1	0	0	0	0	0
c09. The main effect of dot periodicity																			
1	1	1	1	1	-1	-1	-1	-1	-1	-1	-1	-1	-1	-1	1	1	1	1	1

Note that regressors in VMCp and VMCn were made such that each regressor emulated the regressor of each motion speed (stimulus onset and duration) in the tactile classification task. SCP: speed classification-periodic; SCN: speed classification-non-periodic. VMCp: visual motor control-periodic; VMCn: visual motor control-non-periodic.

Supplementary Table 2. Brain regions activated by speed classification of periodic and non-periodic surfaces

Spatial extent test		MNI coordinates			*T* value	Hem	Anatomical region
**Cluster size (mm^3)**	***P*-value**	**x**	**y**	**z**			
Brain regions more strongly activated by SCP than VMCp (SCP - VMCp)							
115792	<0.001	-30	-46	66	6.94	L	Superior parietal lobule
		-50	-28	58	16.22	L	Postcentral gyrus
		-54	8	22	6.97	L	Precentral gyrus
		-48	-22	20	17.65	L	Parietal operculum
		-42	36	18	5.92	L	Middle frontal gyrus
		-38	-18	14	11.77	L	Insula
		-42	42	8	5.66	L	Inferior frontal gyrus
		-12	-24	8	5.25	L	Thalamus
		10	-26	8	3.59	R	Thalamus
		-14	6	-6	5.22	L	Putamen
		-6	-28	-18	5.09	L	Brainstem
		6	-30	-20	4.44	R	Brainstem
88256	<0.001	56	-44	52	5.98	R	Inferior parietal lobule
		36	-2	50	4.31	R	Middle frontal gyrus
		50	8	22	7.88	R	Precentral gyrus
		54	-20	22	12.81	R	Parietal operculum
		44	40	8	8.19	R	Inferior frontal gyrus
		32	22	2	8.62	R	Insula
		52	20	-10	4.58	R	Inferior frontal gyrus
3784	<0.01	-12	-24	42	7.74	L	Cingulate gyrus
18104	<0.001	-12	22	66	3.11	L	Superior frontal gyrus
		6	20	48	7.41	R	Superior frontal gyrus
		-2	4	36	4.23	L	Cingulate gyrus
		6	10	26	3.12	R	Cingulate gyrus
2800	<0.05	16	4	-4	5.13	R	Putamen
2904	<0.05	-18	-78	-50	6.68	L	Cerebellum
7032	<0.001	18	-68	-48	6.65	R	Cerebellum
4440	<0.01	-22	-70	-26	4.95	L	Cerebellum

Brain regions more strongly activated by SCN than VMCn (SCN -VMCn)							
67024	<0.001	-36	-46	66	5.10	L	Superior parietal lobule
		-32	-4	56	3.76	L	Middle frontal gyrus
		-58	-22	46	13.19	L	Postcentral gyrus
		-54	-46	40	3.49	L	Inferior parietal lobule
		-50	-22	20	14.29	L	Parietal operculum (PO)
		-58	6	12	5.83	L	Precentral gyrus
		-40	-6	8	7.81	L	Insula
		-46	12	-8	3.41	L	Inferior frontal gyrus
83176	<0.001	40	2	58	4.27	R	Middle frontal gyrus
		60	-28	48	6.62	R	Inferior parietal lobule
		50	4	42	4.29	R	Precentral gyrus
		56	-18	22	11.41	R	Parietal operculum (PO)
		56	14	10	8.02	R	Inferior frontal gyrus
		32	20	0	8.48	R	Insula
14192	<0.001	6	22	46	7.83	R	Superior frontal gyrus
		-4	22	44	6.55	L	Superior frontal gyrus
		10	16	28	2.87	R	Cingulate gyrus
5848	<0.01	16	-70	-52	6.84	R	Cerebellum
9272	<0.001	-10	-16	8	4.91	L	Thalamus
		8	-14	4	4.64	R	Thalamus
		-14	6	-2	5.08	L	Putamen
4640	<0.01	-42	42	12	4.50	L	Inferior frontal gyrus
2760	<0.05	24	-54	-26	4.33	R	Cerebellum
2536	<0.05	-24	-66	-28	4.23	L	Cerebellum
2536	<0.05	-8	-26	-12	3.86	L	Brainstem
		6	-26	-14	3.95	R	Brainstem

The extent threshold of activation was P < 0.05, FDR corrected for multiple comparisons over the whole brain with height threshold set at P < 0.005 uncorrected (one-tailed). Hem, hemisphere; R: right; L: left.

Supplementary Table 3. Brain regions commonly activated by periodic and non-periodic surfaces (conjunction analysis)

Spatial extent test		MNI coordinates			*T* value	Hem	Anatomical region
Cluster size(mm^3)	*P*-value	X	y	z			
Common activation between SCP - VMCp and SCN - VMCn (Conjunction analysis of SCP - VMCp and SCN - VMCn)							
64624	< 0.001	-36	-46	66	5.10	L	Superior parietal lobule
		-36	-4	62	3.76	L	Precentral gyrus
		-58	-22	46	13.19	L	Postcentral gyrus
		-50	-22	20	14.29	L	Parietal operculum
		-40	-6	8	7.81	L	Insula
72744	< 0.001	62	-26	40	7.04	R	Inferior parietal lobule
		56	-18	22	11.41	R	Parietal operculum
		58	12	14	7.52	R	Inferior frontal gyrus
		32	22	0	8.39	R	Insula
11352	< 0.001	-4	22	44	6.43	L	Superior frontal gyrus
		6	20	48	7.41	R	Superior frontal gyrus
4856	< 0.01	16	-70	-52	6.46	R	Cerebellum
		30	-50	-50	3.61	R	Cerebellum
1896	< 0.05	-14	6	-4	4.94	L	Putamen
3976	< 0.01	-12	-18	10	4.72	L	Thalamus
		10	-16	12	3.36	R	Thalamus
4600	< 0.01	-42	42	12	4.50	L	Inferior frontal gyrus
2120	< 0.05	-24	-68	-28	4.19	L	Cerebellum
1880	< 0.05	20	-66	-22	4.16	R	Cerebellum
Common activation between VMCp - SCP and VMCn - SCN (Conjunction analysis of VMCp - SCP and VMCn - SCN)							
52176	<0.001	-6	-38	38	5.16	L	Cingulate gyrus
		26	-76	32	5.32	R	Superior occipital gyrus
		-44	-74	30	4.35	L	Angular gyrus
		-16	-84	28	4.80	L	Superior occipital gyrus
		-10	-58	20	4.18	L	Precuneus
		36	-76	6	4.87	R	Middle occipital gyrus
		-36	-78	2	3.73	L	Middle occipital gyrus
		42	-62	-4	5.58	R	Fusiform gyrus

		-46	-56	-12	4.11	L	Inferior temporal gyrus
		38	-48	-18	4.25	R	Fusiform gyrus
		-4	-66	-22	4.46	L	Cerebellum
17248	<0.001	46	-14	54	5.31	R	Postcentral gyrus
		38	-16	42	6.21	R	Precentral gyrus
12832	<0.001	-16	56	16	3.39	L	Middle frontal gyrus
		6	56	14	5.34	R	Superior frontal gyrus
		-6	52	10	5.66	L	Superior frontal gyrus

The extent threshold of activation was $P < 0.05$, FDR corrected for multiple comparisons over the whole brain with the height threshold set at $P < 0.005$ uncorrected (one-tailed). Hem, hemisphere; R, right; L, left. Conjunction analysis was conducted with a conjunction-null hypothesis (Nichols et al., 2005).

Supplementary Table 4 F tests on main effects of speed and periodicity and their interaction.

Spatial extent test		MNI coordinates			*F* value	Hem	Anatomical region
Cluster size (mm^3)	*P*-value	x	y	z			
							The interaction effects between speed and dot periodicity
							No significant activation
							The main effect of speed
384888	< 0.001	-26	-50	66	10.38	L	Superior parietal lobule
		22	-46	70	9.36	R	Superior parietal lobule
		-26	-16	68	11.32	L	Precentral gyrus
		52	0	4	10.19	R	Precentral gyrus
		-44	-30	60	13.88	L	Postcentral gyrus
		32	-28	62	9.19	R	Postcentral gyrus
		-6	-8	56	11.26	L	Superior frontal gyrus
		16	2	68	12.67	R	Superior frontal gyrus
		-32	-82	42	7.12	L	Angular gyrus
		50	-38	20	9.53	R	Angular gyrus
		-6	-74	34	12.98	L	Precuneus
		8	-72	42	10.81	R	Precuneus
		-46	-26	32	7.66	L	Supramarginal gyrus
		54	-20	44	9.01	R	Supramarginal gyrus
		26	-80	38	5.2	R	Superior occipital gyrus
		-4	-28	30	15.97	L	Posterior cingulate gyrus
		6	-24	40	19.35	R	Posterior cingulate gyrus
		-60	-22	16	15.64	L	Parietal operculum
		52	-22	20	14.38	R	Parietal operculum
		-6	-70	16	3.72	L	Cuneus
		4	-78	10	7.16	R	Cuneus
		-52	-70	10	9.43	L	Middle occipital gyrus
		38	-76	2	7.81	R	Middle occipital gyrus
		-30	-22	0	5.07	L	Putamen
		28	-12	10	4.09	R	Putamen
		-50	-68	8	9.24	L	Middle temporal gyrus
		44	-50	6	8.89	R	Middle temporal gyrus

		48	0	4	10.17	R	Inferior frontal gyrus
		-40	-4	4	11.81	L	Insula
		40	0	-10	13.4	R	Insula
		-18	24	-4	8.15	L	Caudate nucleus
		10	18	8	6.15	R	Caudate nucleus
		-26	-36	0	13.99	L	Hippocampus
		34	-22	-14	13.97	R	Hippocampus
		30	42	22	9.22	R	Middle frontal gyrus
		-18	-34	-8	10.8	L	Parahippocampal gyrus
		16	-32	-2	14.32	R	Parahippocampal gyrus
		-50	4	-4	7.28	L	Superior temporal gyrus
		48	12	-10	7.83	R	Superior temporal gyrus
		-14	44	-6	13.95	L	Medial prefrontal cortex
		6	52	-6	9.26	R	Medial prefrontal cortex
		-28	-6	-12	5.17	L	Amygdala
		22	-2	-14	7.89	R	Amygdala
		-24	32	-16	8.11	L	Orbitofrontal gyrus
		22	30	-8	6.72	R	Orbitofrontal gyrus
		-48	-56	-16	9.2	L	Inferior tempooral gyrus
		48	-46	-12	7.9	R	Inferior tempooral gyrus
		-2	-82	-16	6.92	L	Lingual gyrus
		8	-82	-14	7.46	R	Lingual gyrus
		-40	-42	-26	5.49	L	Fusiform gyrus
		40	-42	-14	7.82	R	Fusiform gyrus
		-18	-84	-20	7.92	L	Inferior occipital gyrus
		32	-76	-18	12.24	R	Inferior occipital gyrus
		-42	-58	-26	10.41	L	Cerebellum
		36	-52	-26	12.91	R	Cerebellum
		-18	-38	-38	6.73	L	Brainstem
		22	-22	-10	9.8	R	Brainstem
4144	0.011	-36	42	22	9.4	L	Middle frontal gyrus
3288	0.023	30	24	0	6.09	R	Insula
		42	36	10	5.57	R	Inferior frontal gyrus
		The main effect of dot periodicity					
5208	0.009	-42	-24	54	13.76	L	Postcentral gyrus
		-22	-42	68	11.74	L	Superior parietal lobule

The extent threshold of activation was $P < 0.05$, FDR corrected for multiple comparisons over the whole brain with the height threshold set at $P < 0.01$ (uncorrected). Hem, hemisphere; R, right; L, left.

Supplementary Table 5 Brain activation related to motion speed (t contrasts)

Spatial extent test		MNI coordinate			*T* value	Hem	Anatomical region
Cluster size (mm^3)	*P*-value	X	y	Z			
Brain regions of which activity was positively related to motion speed (Speed+, Figure 4)							
4680	<0.01	-6	22	44	4.04	L	Superior frontal gyrus
		6	20	50	4.37	R	Superior frontal gyrus
6344	<0.05	36	26	6	4.63	R	Inferior frontal gyrus
		36	16	-6	3.23	R	Insula
3160	< 0.05	-40	-18	18	4.32	L	Parietal operculum (PO)
Brain regions of which activity was negatively related to motion speed (Speed–)							
15192	< 0.001	32	-28	50	5.04	R	Postcentral gyrus
		26	-18	72	4.09	R	Precentral gyrus
10344	< 0.001	-16	-52	-20	5.66	L	Cerebellum

The extent threshold of activation was $P < 0.05$, FDR corrected for multiple comparisons over the whole brain with height threshold set at $P < 0.005$ uncorrected (one-tailed). Hem, hemisphere; L, left; R, right.

Supplementary Table 6 Differences in brain activation between periodic and non-periodic surfaces (t contrasts)

Spatial extent test		MNI coordinate			*T* value	Hem	Anatomical region
Cluster size (mm^3)	*P*-value	X	y	z			
Brain regions more strongly activated by SCP than SCN (Figure 5) [(SCP - VMCp) - (SCN - VMCn)]							
5208	<0.01	-22	-42	68	3.43	L	Superior parietal lobule
		-42	-24	54	3.71	L	Postcentral gyrus
Brain regions more strongly activated by SCN than SCP [(SCN - VMCn) - (SCP - VMCp)]							
No significant activation							

The extent threshold of activation was $P < 0.05$, FDR corrected for multiple comparisons over the whole brain with height threshold set at $P < 0.005$ uncorrected (one-tailed). Hem, hemisphere; L, left.

Supplementary Table 7 Psycho-physiological interaction (PPI) analysis

Spatial extent test		MNI coordinate			*T* value	Hem	Anatomical region
Cluster size (mm³)	*P*-value	x	y	z			
Psycho-physiological interaction (PPI) analysis with the left postcentral gyrus as a seed region (Figure 6)							
2024	< 0.05	-32	16	4	4.98	L	Insula
		-28	24	-12	5.77	L	Lateral orbitofrontal gyrus
5160	<0.01	-52	-26	50	3.97	L	Postcentral gyrus
		-48	-50	46	5.04	L	Inferior parietal lobule
		-34	-48	38	3.98	L	Superior parietal lobule
2240	<0.05	-34	0	34	3.69	L	Middle frontal gyrus
		-46	8	26	4.99	L	Inferior frontal gyrus
2864	<0.05	-50	-18	20	4.82	L	Parietal operculum (PO)
1944	<0.05	56	10	38	3.98	R	Precentral gyrus
		52	12	30	4.52	R	Inferior frontal gyrus
3936	< 0.01	24	-72	2	4.28	R	Lingual gyrus
		40	-64	-18	3.83	R	Inferior occipital gyrus
		44	-56	-24	3.37	R	Fusiform gyrus
		30	-66	-32	3.89	R	Cerebellum

The extent threshold of activation was $P < 0.05$, FDR corrected for multiple comparisons over the whole brain with height threshold set at $P < 0.005$ uncorrected (one-tailed). Hem, hemisphere; L, left; R, right.

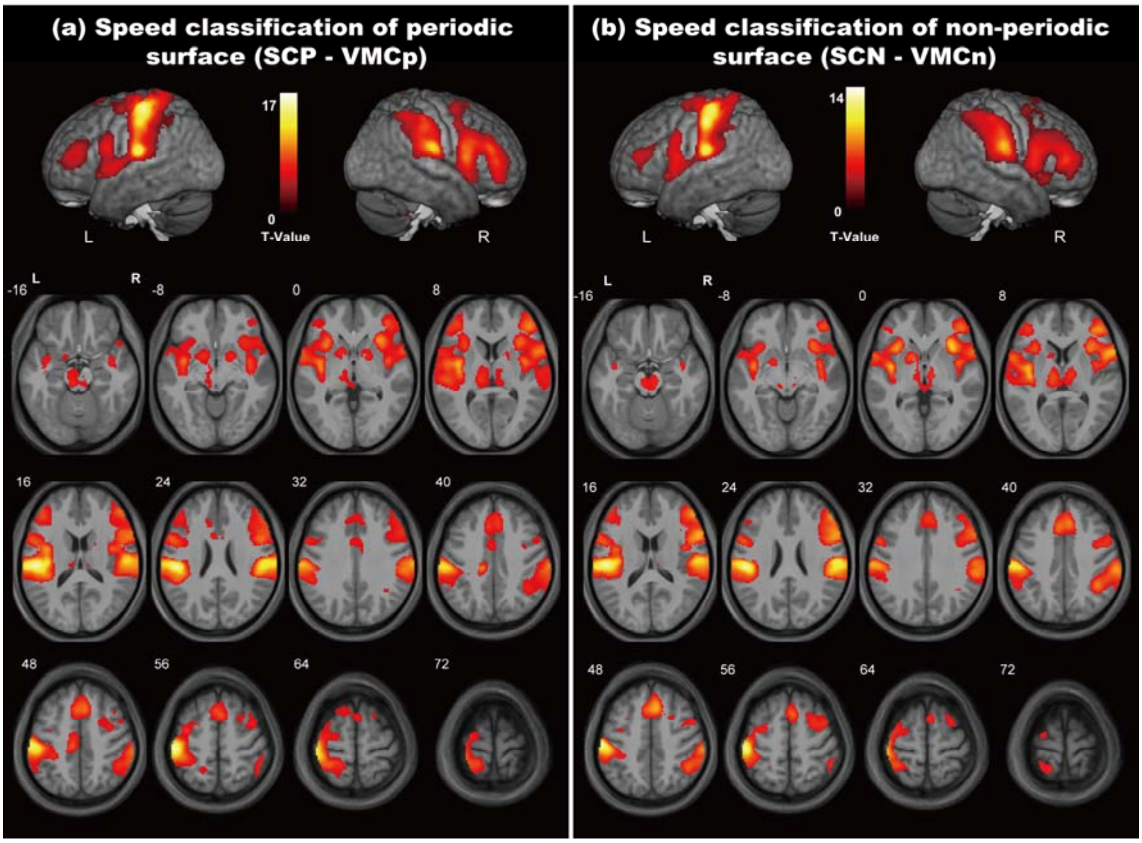

Supplementary Figure 1. Brain activation during speed classification tasks (relative to visual motor control, VMC)

The results of the contrast of SCP–VMCp and SCN–VMCn are depicted above. Areas of significant activation are rendered and superimposed on the T1-weighted MRI averaged across the participants. Each number located at the top left of each horizontal slice indicates z coordinate (from -16 to 72). The extent threshold of activation was $P < 0.05$, FDR corrected for multiple comparisons over the whole brain with the height threshold set at $P < 0.005$ uncorrected (one-tailed). R, right; L, left.

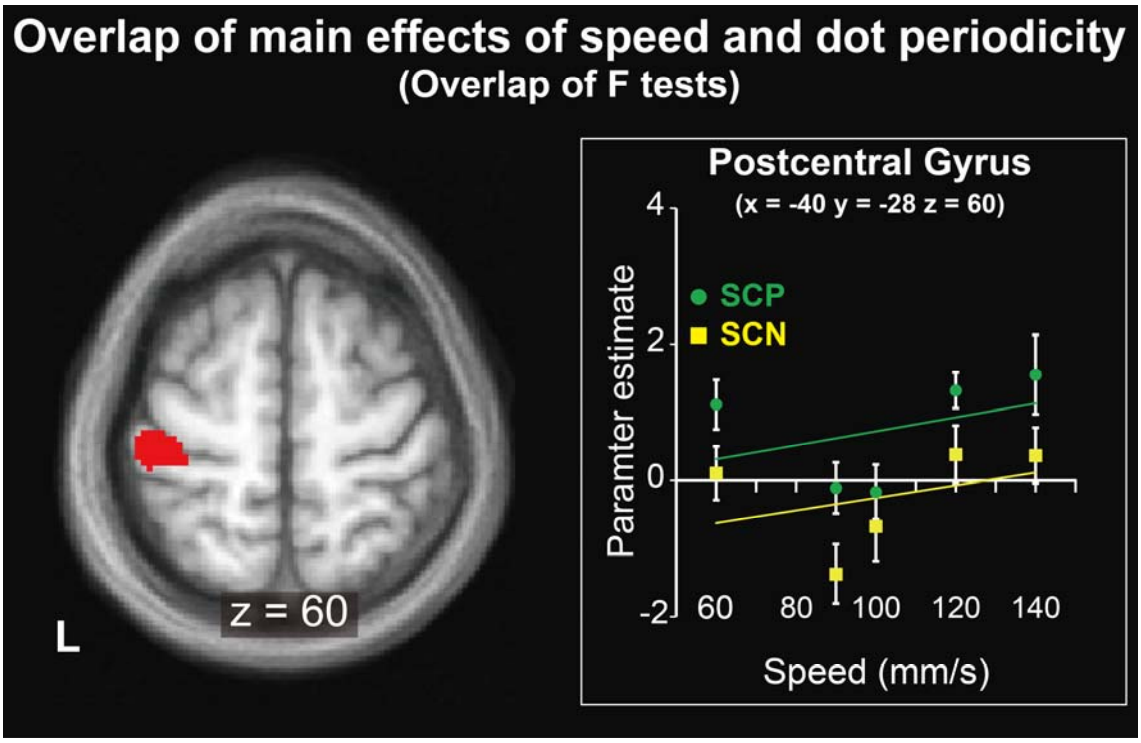

Supplementary Figure 2. Overlap of main effects of speed and dot periodicity

Overlap of activation between main effects of speed and dot periodicity, which were evaluated by the F contrasts. Two-way repeated measures ANOVA (5 levels of speed ×2 levels of dot periodicity) of activity in the center of mass of overlap (x = - 48, y = -28, z = 60) showed both main effects of periodicity ($F(1, 19) = 10.2$, $p = 0.005$) and speed ($F(4, 76) = 13.1$, $p < 0.001$) without interactions ($P = 0.67$). One sample t tests showed that mean fitted slopes of linear functions were significantly greater than zero ($t(19) = 2.23$ $p = 0.04$ for periodic and $t(19) = 2.36$ $p = 0.03$ for non-periodic surfaces). Areas of significant activation are superimposed on the T1-weighted MRI averaged across the participants. The extent threshold of activation was $P < 0.05$, FDR corrected for multiple comparisons over the whole brain with the height threshold set at $P < 0.01$ (uncorrected). R, right; L, left.

References

1. Nichols, T., Brett, M., Andersson, J., Wager, T. & Poline, J. B. Valid conjunction inference with the minimum statistic. NeuroImage 25, 653-660 (2005).
